# Smoking cessation during pregnancy: a population-based study

**DOI:** 10.11606/S1518-8787.2019053000619

**Published:** 2018-12-20

**Authors:** Josiane Luzia Dias-Damé, Ana Cristina Lindsay, Juraci Almeida Cesar

**Affiliations:** I Universidade Federal de Pelotas. Faculdade de Odontologia. Departamento de Odontologia Social e Preventiva. Pelotas, RS, Brasil; II University of Massachusetts Boston. College of Nursing and Health Sciences. Department of Exercise and Health Sciences. Boston, MA, EUA; III Harvard T.H. Chan School of Public Health. Department of Nutrition. Boston, MA, EUA; IV Universidade Federal de Pelotas. Faculdade de Medicina. Departamento de Medicina Social. Programa de Pós-Graduação em Epidemiología. Pelotas, RS, Brasil; V Universidade Federal do Rio Grande. Faculdade de Medicina. Programa de Pós-Graduação em Saúde Pública. Rio Grande, RS, Brasil

**Keywords:** Pregnant Women, Tobacco Use Cessation, Maternal and Child Health, Cross-Sectional Studies, Gestantes, Abandono do Uso de Tabaco, Saúde Materno-Infantil, Estudos Transversais

## Abstract

**OBJECTIVE:**

To measure the prevalence of smoking cessation during pregnancy and to identify factors associated with its occurrence.

**METHODS:**

The present survey included all puerperal women living in the municipality of Rio Grande, RS, whose birth occurred between January 1 and December 31, 2013. A single standardized questionnaire was applied, in the hospital, within 48 hours of delivery. Multivariate analysis was performed using Poisson regression with robust variance.

**RESULTS:**

The prevalence of smoking cessation among the 598 parturients studied was 24.9% (95%CI 21.5-28.6). After adjusting for confounding factors, mothers aged 13 to 19 years (PR = 1.76; 95%CI 1.13-2.74), who had higher family income (PR = 1.83; 95%CI, 1.23-2.72), higher educational level (PR = 2.79; 95%CI 1.27-6.15), higher number of prenatal appointments (PR = 1.84; 95%CI 1.11-3.05), and who did not smoke in the previous pregnancy (PR = 2.93; 95% CI, 1.95-4.41) presented a higher prevalence ratio of smoking cessation.

**CONCLUSIONS:**

Although pregnancy is a window of opportunity for smoking cessation, the rate of cessation was low. The prevalence of cessation was higher among mothers with lower risk of complications, suggesting the need for interventions prioritizing pregnant women of lower socioeconomic levels.

## RESUMO

**OBJETIVO:**

Medir a prevalência de cessação do tabagismo durante a gestação e identificar fatores associados à sua ocorrência.

**MÉTODOS:**

O presente inquérito incluiu todas as puérperas residentes no município de Rio Grande, RS, cujo parto tenha ocorrido entre primeiro de janeiro e 31 de dezembro de 2013. Aplicou-se um questionário único padronizado no hospital em até 48 após o parto. A análise multivariada foi feita por meio da regressão de Poisson com variância robusta.

**RESULTADOS:**

A prevalência de cessação do tabagismo entre as 598 parturientes estudadas foi de 24,9% (IC95% 21,5-28,6). Após ajuste para fatores de confusão, mães com idade entre 13 e 19 anos (RP = 1,76; IC95% 1,13-2,74), maior renda familiar (RP = 1,83; IC95% 1,23-2,72), maior escolaridade (RP = 2,79; IC95% 1,27-6,15), maior número de consultas de pré-natal (RP = 1,84; IC95% 1,11-3,05) e que não fumaram na gestação anterior (RP = 2,93; IC95% 1,95-4,41) apresentaram maior razão de prevalências de cessação do tabagismo que as demais.

**CONCLUSÕES:**

Apesar de a gestação ser um momento propício à interrupção do tabagismo, foi baixa a proporção de cessação. Verificou-se ainda que a prevalência de cessação foi maior entre mães com menor risco de complicações. Isso evidencia a necessidade de intervenções continuadas priorizando gestantes de pior nível socioeconómico.

## INTRODUCTION

Smoking during pregnancy is the main preventable risk factor for several unfavorable outcomes, both for the baby and the pregnant woman. Smoking accounts for 5% to 8% of premature births, 13% to 19% of cases of low birth weight, and 5% to 7% of sudden infant death syndrome^1^. Thus, reducing the occurrence of smoking during pregnancy is a public health priority. Considering all the moments in which smoking can be discouraged, the gestational period is possibly the one with greatest potential impact. Knowledge of the possibility of causing harm to the fetus, frequent contact between pregnant woman and health service, and the social definition of this practice as reprehensible can contribute to the reduction in the number of cigarettes smoked and to smoking cessation. Since the mother wants, above all else, the well-being of her child, she will be much more willing to make a more forceful effort than in other situations where smoking is normally discouraged. Reducing the intensity of exposure to smoke brings benefits to both mother and newborn, and the earlier this occurs, the lower the adverse effects^2^ ^,^ ^3^.

Most of the existing studies on the subject have been conducted in developed countries and show that smoking cessation in the gestational period is more common among primiparous women, who live with their partners, have higher income and schooling, have planned their pregnancies, and used to smoke fewer cigarettes per day^4^ ^-^ ^9^. In two cohort studies conducted in Pelotas, RS, in 1982 and 1993, a direct relationship was observed between the number of prenatal consultations and the rate of smoking cessation. In both periods, smoking cessation during pregnancy was more common among women with better socioeconomic level^5^. In a study carried out in two public maternity hospitals in the state of Rio de Janeiro, in 2011, the observed cessation prevalences were 28% and 32%^10^. In a study conducted in Porto Alegre, RS, which included only women who underwent prenatal care, 25% of the parturients were classified as smokers in abstinence^11^.

Most studies address smoking cessation at defined periods, without considering pregnancy as a whole. However, women who continue to smoke during pregnancy may temporarily stop smoking, reduce the number of cigarettes, and have multiple relapses during the gestational period. Pickett et al.^7^ observed that about 40% of pregnant women underwent at least one attempt to quit smoking, with only half of them remaining abstinent until the end of pregnancy. In 2010, Motta et al. showed that 45% of pregnant smokers stopped smoking and relapsed during pregnancy^11^.

In addition to the lack of population-based studies on smoking cessation during pregnancy carried out in low- and middle-income countries, there is a lack of knowledge on cessation attempts, as well as on the identification of withdrawal periods during pregnancy.

This study aimed to describe smoking cessation during pregnancy, to estimate its prevalence and to investigate associated factors.

## METHODS

The data used in this analysis were collected as part of a larger study conducted every three years in Rio Grande, a municipality located in the so-called South Half of the state of Rio Grande do Sul. In 2013, the municipality had about 206 thousand inhabitants, according to an estimate by the Brazilian Institute of Geography and Statistics.

The target population was comprised of women living in Rio Grande's urban and rural areas who had children in one of the two local maternities between January 1 and December 31, 2013. These maternities belong to Dr. Miguel Riet Corrêa Jr., from the Universidade Federal do Rio Grande and the Santa Casa de Misericórdia. Parturients whose newborns were weighted at less than 500 grams or born after less than 20 weeks’ pregnancy were excluded from the study. This criterion was adopted because the World Health Organization classifies these situations as fetal mortality, excluding them from perinatal statistics.

All information presented in this article was obtained through the application by previously trained interviewers of a single standardized questionnaire, within 48 hours of delivery. The pilot study was conducted in 2012, in the same maternities, with the objective of testing the enunciation of questions and the logistics of data collection.

The interviewers visited the delivery room of each of the maternities on a daily basis, checked the hospitalization registry book, and then confirmed the obtained information in each hospital's Medical and Statistical Service (SAME). Afterwards, they wrote down the mother's name and went to the infirmary. After confirming with the mother that she in fact resided in the municipality of Rio Grande, they explained the purpose of the study, inviting her to participate.

The completed questionnaires were later coded by the interviewer and delivered to the project headquarters, where they were reviewed and typed into the Epidata 3.1 program^12^, twice, by different professionals, with the second typing in the reverse order of the first one. Each block of 100 typed questionnaires was then compared, and any differences were corrected. Quality control was performed by a single person, via telephone, through the application of a reduced questionnaire containing key questions. This involved 7% of the respondents. Regarding smoking, the following question was included: “In the six months prior to this pregnancy, did you smoke?” The responses were compared using the Kappa agreement test. This resulted in a value of 0.63, considered a moderate level of agreement.

Information on smoking six and three months before becoming pregnant and also during each quarter of pregnancy was collected. Parturients who reported smoking six or three months prior or during pregnancy were included in the study. In addition, we investigated whether these women attempted to quit smoking, how many attempts were made, when they occurred and for how many days they went without smoking. Based on these data, the “cessation of smoking” outcome was designed and attributed to women who smoked six or three months prior to pregnancy, stopped smoking before the seventh month, and did not return to smoking until the end of pregnancy. Thus, only those who reported smoking six or three months before pregnancy were included in the crude and adjusted analyzes. The basis for determining the sixth month of pregnancy as the limit for cessation were studies which demonstrate a reduction in the risk of adverse effects when the cessation occurs within the first^13^ ^,^ ^14^ or second quarter^15^.

The following exposure variables, collected whenever possible in the continuous or discrete forms, were included in the analysis model: maternal age (13-19, 20-24, 25-29, 30 years or more), cohabitation with partner (yes, no), schooling (1-4, 5-8, 9 years or more), monthly family income (tertiles), work during pregnancy (yes, no), parity (primiparous, multiparous), preterm birth (yes, no), start of prenatal care (quarter), number of prenatal consultations performed (1-5, 6 or more), type of service in which the prenatal consultations were performed (public or private), support received from the child's father during pregnancy assessed as positive (yes, no), husband/partner smoked for at least one quarter during pregnancy (yes, no), number of cigarettes smoked per day before pregnancy (1-10, 11-20, 21 or more), and smoking in the previous pregnancy (yes, no).

Descriptive analysis consisted in obtaining measures of prevalence for both the exposure and outcome variables. Pearson's chi-square test or Fisher's exact test were used for comparing proportions between independent variables and outcome^16^. Variables shown to have p ≤ 0.20 in the bivariate analysis were carried forward to the adjusted analysis. The only exception was the “maternal age” variable, which, in addition to having fundamental importance for maternal and child health, presented a p-value (p = 0.235) very close to the previously mentioned limit. For these reasons it was included in the model and all other variables were adjusted to it.

Adjusted analysis, carried out with the objective of controlling potential confounding factors, obeyed a previously established hierarchical model^17^. This model, proposed by Victora et al., is divided into outcome levels (distal, intermediate and proximal), and assumes that variables located at the hierarchically superior level are determinants of those at lower levels^17^. Thus, variables at the distal level exert influence over (and even determine) those at the intermediate and proximal levels, while intermediate variables may exert influence over those at the proximal level. Variables located at a hierarchically superior level to that of the variable under analysis are considered potential confounders in the relationship between the exposure under testing and the outcome. Meanwhile, variables located at lower levels are considered as potential mediators of this association.

The model proposed for this analysis has three levels. The first level is comprised of variables related to the mother's demographic characteristics, as well as the family's socioeconomic characteristics (maternal age, cohabitation with the partner, family income, maternal schooling and mother working during pregnancy); the second level is comprised of variables related to reproductive history (history of preterm birth and parity); the third level is comprised of variables related to the current pregnancy and previous exposure to smoking (start of prenatal care in the first quarter, the type of service in which prenatal care was performed, number of prenatal consultations, support of the father, whether the partner is a smoker, number of cigarettes smoked prior to pregnancy, and whether the mother smoked during the previous pregnancy). In this regression model, the variables are controlled using those at the same or previous levels. However, it was decided *a priori* that, in order to be kept in the model, the p-value of the association between the exposure variable and the outcome should be ≤ 0.20. The level of significance for the two-tailed tests was 5%.

The measure of effect used was prevalence ratio (PR), obtained by means of Poisson regression with robust variance adjustment^18^. For ordinal categorical variables, the p-value of the linear trend test was reported. For the other variables, the Wald test was employed to assess heterogeneity^16^. All analyzes were performed using the Stata statistical package version 11.2^19^.

This research project was submitted and approved by the Research Ethics Committee of the Federal University of Rio Grande and by the Committee of Ethics in Health Research of Rio Grande's Santa Casa. The free and informed consent term was read by each participant, who signed two copies, one remaining in the participant's possession. In the case of adolescent parturients who agreed to participate in the study, the term was preferably signed by the spouse or by one of the parents. In addition, the right of non-participation in the research was protected, as well as the confidentiality of the information obtained.

## RESULTS

The 2013 Perinatal Study identified 2724 eligible parturients and interviewed 2653. Losses were 71 (2.6%) parturients in total. Among all interviewees, 749 (28.2%; 95%CI 26.5-29.9) had a history of current or previous smoking. One hundred and forty-eight were excluded, since they had stopped smoking prior to the six months preceding the current pregnancy. Three were excluded because they started smoking during pregnancy. Thus, the study's denominator was comprised of 598 puerperae.

Table 1 shows that the majority of mothers were between 20 and 29 years old (55%), lived with their partner (79%), had between five and eight years of schooling (52%), were multiparous (77%), started prenatal care in the first quarter (67%), and had more than five prenatal consultations (77%), predominantly in the public health service (71%). Finally, 68% of parturients smoked in the previous pregnancy and 49% smoked 11 to 20 cigarettes in the six months prior to pregnancy.

**Table 1:** Distribution of the studied population according to demographic, socioeconomic and maternal characteristics. Rio Grande, state of Rio Grande do Sul, 2013. (n = 598)

Characteristic		n	%
Maternal age (years)			
	13 to 19	83	13.9
	20 to 24	174	29.1
	25 to 29	153	25.6
	30 or more	188	31.4
	Cohabitation with partner	474	79.3
Income (tertiles)^a^			
	1 (poorest)	210	35.1
	2	188	31.5
	3 (richest)	200	33.4
Schooling (full years)			
	1 to 4	63	10.5
	5 to 8	311	52.0
	9 or more	224	37.5
	Worked during pregnancy	202	33.8
	Previous preterm birth (n = 462)	80	17.3
	Primiparous	136	22.7
	Performed prenatal consultations in the public health service (n = 560)	397	71.0
	Started prenatal care in the first quarter (n = 559)^b^	375	67.1
	Received support from the father of the child during pregnancy	484	80.9
	Had 6 or more consultations (n = 560)	430	76.8
	Smoker partner	310	51.8
	Smoked in the previous pregnancy (n = 442)^c^	302	68.3
Cigarettes/day before pregnancy			
	1-10	220	36.8
	11-20	295	49.3
	21 or more	83	13.9

a Tertile 1 (poorest): 0 to 1.5 minimum wages; Tertile 2: 1.5 to 2.6; Tertile 3 (richest): 2.7 to 17.7. Minimum wage in 2013: R$678.00.

b Lack of information on one woman.

c Lack of information on 20 women.

The Figure shows smoking cessation from the previous six months to the end of pregnancy. It is noteworthy that 595 women used to smoke in the six months prior to pregnancy, and 3 started smoking in the three months prior to pregnancy. Among the 34 women who stopped smoking in the six months prior to pregnancy but then resumed smoking, 24 did so between the third quarter and the immediate postpartum period. Prevalence of cessation was higher between the three months prior to pregnancy and the first quarter of pregnancy (17%) and between the first and second quarters (11%), decreasing as the pregnancy progressed: in the third quarter only two pregnant women stopped smoking.

**Figure:**
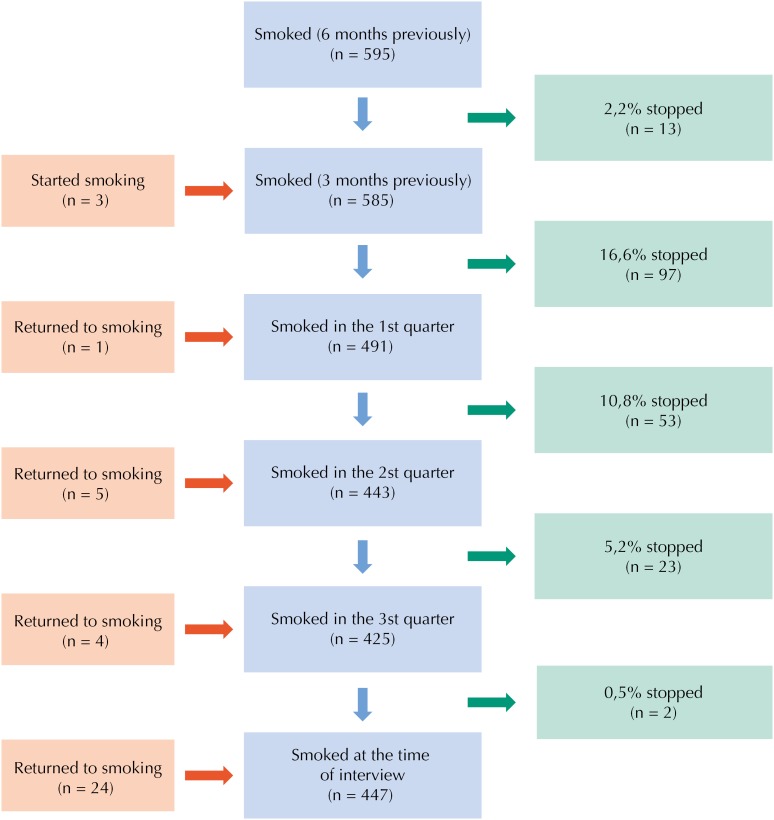
Description of smoking cessation in the gestational period among puerperal women living in the municipality of Rio Grande, state of Rio Grande do Sul, Brazil.

According to data on attempted cessation, 56% of women attempted to quit smoking, 78% of whom went without smoking for at least seven consecutive days. The first attempt occurred more frequently (44%) in the first quarter of pregnancy. In addition, most women made only one attempt to stop smoking (77%). Overall prevalence of cessation was 24.9% (95%CI 21.5-28.6) (Table 2). Table 3 shows the prevalence of smoking cessation by category of variable included in the model. It ranged from 9.5% (among women with one to four years of schooling) to 44.3% (among women who did not smoke in the previous pregnancy). In the crude analysis, a higher PR was observed among women who underwent prenatal care in the private service (PR = 1.46; 95%CI 1.10-1.94). After adjustment, however, this association was reversed, with a lower PR among those who performed the consultations in the private service (PR = 0.61; 95%CI 0.39-0.95). Other variables remained significantly associated with smoking cessation after adjustment, including maternal age: adolescent mothers had PR = 1.76 (95%CI 1.13-2.74) when compared to women with 30 years of age or more. In respect to family income and mother's schooling, a higher prevalence ratio was found among women with higher income (PR = 1.83; 95%CI 1.23-2.72) and schooling (PR = 2.79; 95%CI 1.27-6.15), when compared to those with lower income and only one to four years of schooling. Prevalence rate was also higher among women who underwent six or more prenatal consultations (PR = 1.84; 95%CI 1.11-3.05), and among those who did not smoke in the previous pregnancy (PR = 2.93, 95%CI 1.95-4.41).

**Table 2:** Attempts of smoking cessation and actual smoking cessation during pregnancy. Rio Grande, state of Rio Grande do Sul, 2013. (n = 598)

Variable		n	%
Tried to quit smoking		334	55.9
Number of attempts			
	1	258	77.2
	2	42	12.6
	3 or +	34	10.2
Moment of the first cessation attempt*			
	Before pregnancy	102	30.6
	1st quarter	147	44.2
	2nd and 3rd quarters	84	25.2
Total days without smoking			
	0-6	73	21.9
	7-30	59	17.7
	31-90	29	8.6
	> 90	173	51.8
	Cessation before the 3rd quarter	149	24.9

* Absence of data on one parturient.

**Table 3:** Prevalence by category; crude and adjusted analysis of smoking cessation during pregnancy; associated factors. Rio Grande, state of Rio Grande do Sul, 2013. (n = 598)

Variable		Prevalence of cessation(%)	Crude PR (CI)	Adjusted PR (CI)
Level I				
Demographic				
Maternal age (years)			0.235^a^	0.010^a^
	13 to 19	28.9	1.29 (0.84-1.99)	1.76 (1.13-2.74)
	20 to 24	25.9	1.16 (0.80-1.67)	1.37 (0.96-1.95)
	25 to 29	24.8	1.11 (0.76-1.63)	1.15 (0.79-1.67)
	30 or more	22.3	1.00	1.00
Cohabitation with partner			0.028	0.058
	No	16.9	1.00	1.00
	Yes	27.0	1.59 (1.05-2.42)	1.50 (0.99-2.28)
Socioeconomic				
Income (tertiles)^b^			< 0.001^a^	0.003^a^
	1 (poorest)	15.2	1.00	1.00
	2	26.6	1.74 (1.1 7-2.60)	1.56 (1.05-2.31)
	3 (richest)	33.5	2.20 (1.51-3.20)	1.83 (1.23-2.72)
Schooling (full years)			< 0.001^a^	0.001^a^
	1 to 4	9.5	1.00	1.00
	5 to 8	22.5	2.36 (1.07-5.20)	2.12 (0.96-4.64)
	9 or +	32.6	3.42 (1.56-7.50)	2.79 (1.27-6.15)
Worked during pregnancy			0.019	0.185
	No	22.0	1.00	1.00
	Yes	30.7	1.40 (1.06-1.85)	1.22 (0.91-1.64)
Level II				
Reproductive history				
Parity			0.061	0.883
	Primiparous	30.9	1.33 (0.99-1.80)	1.02 (0.74-1.41)
	Multiparous	23.2	1.00	1.00
Previous preterm birth (n = 462)			0.072	0.100
	No	24.9	1.00	1.00
	Yes	15.0	0.60 (0.35-1.05)	0.63 (0.36-1.09)
Level III				
Current pregnancy				
Started prenatal care in the first quarter (n = 560)^c^			0.014	0.963
	Yes	29.1	1.53 (1.09-2.14)	1.01 (0.66-1.55)
	No	19.0	1.00	1.00
Type of service for prenatal consultations (n = 560)^c^			0.009	0.028
	Public	22.7	1.00	1.00
	Private	33.1	1.46 (1.10-1.94)	0.61 (0.39-0.95)
Number of consultations (n = 560)^c^			0.001	0.019
	1-5	13.1	1.00	1.00
	6 or more	29.5	2.26 (1.42-3.60)	1.84 (1.1 1-3.05)
Support of the child's father			0.206	0.794
	Yes	26.0	1.29 (0.87-1.92)	1.07 (0.64-1.79)
	No	20.2	1.00	1.00
Smoker partner			0.082	0.239
	Yes	21.9	1.00	1.00
	No	28.1	1.28 (0.97-1.70)	1.23 (0.87-1.74)
Prior exposure to smoking				
Cigarettes/day before pregnancy			< 0.001	0.071
	1-10	37.7	2.61 (1.50-4.53)	1.46 (0.79-2.67)
	11-20	18.3	1.27 (0.71-2.25)	0.95 (0.52-1.73)
	21 or more	14.5	1.00	1.00
Smoking during previous pregnancy (n = 442)			< 0.001	< 0.001
	Yes	12.2	1.00	1.00
	No	44.3	3.61 (2.53-5.15)	2.93 (1.95-4.41)

a p-value trend.

b Tertile 1 (poorest): 0 to 1.5 minimum wages; Tertile 2: 1.5 to 2.6; Tertile 3 (richest): 2.7 to 17.7. Minimum wage in 2013: R$678.00.

c Thirty-eight women who did not perform prenatal care were excluded.

Equation I: age + cohabitation with partner + income + schooling + worked during pregnancy (1st level).

Equation II: Equation I + history of previous preterm birth (2nd level).

Equation III: Equation II + type of service for prenatal consultations + number of prenatal consultations + number of cigarettes smoked prior to pregnancy + smoking in previous pregnancy (3rd level).

## DISCUSSION

This study showed that in the period between six months prior to pregnancy and immediate postpartum 56% of women tried to quit smoking and 78% went without smoking for at least seven consecutive days. Only one in four quit smoking during the gestational period. Cessation was significantly associated with lower age and higher maternal schooling, higher family income, higher number of prenatal consultations, especially in the private sector, and absence of smoking in previous pregnancy.

In the studied sample, 56% of the women reported having tried to quit smoking, a prevalence higher than the 38% observed by Pickett et al.^7^ However, these authors assessed only the gestational period and considered as attempts to quit only cases when the woman went without smoking for at least seven days. They also observed that the first attempt at cessation occurred more frequently in the first quarter of pregnancy (71%). Although our study included an evaluation of the pre-gestational period, it also showed that the first attempt occurs more frequently during the first quarter (44%).

The prevalence of cessation in this study was 25%, similar to the one observed by a study conducted in Porto Alegre, state of Rio Grande do Sul, which included only women who underwent prenatal care, and classified 25% of the participants as abstinent smokers^11^. The prevalence of smoking cessation observed in Rio Grande, state of Rio Grande do Sul, was expected to be higher than that found in Pelotas, state of Rio Grande do Sul, in 1993 (21%)^5^, because, despite the proximity between municipalities, the employed definitions of cessation were not the same. In the study by Horta et al.^5^, the cessation rate considered only on women who used to smoke in the beginning of the pregnancy. In addition, the two studies dealt with very different periods, with a 20-year interval between one another.

In a 2011 study conducted at two public maternity hospitals in the state of Rio de Janeiro, in one of the maternities the prevalence of cessation was greater from one month before pregnancy until the first quarter (18%), and from the first to the second quarter (13%) in the other^10^. Although an interval of three months previous to pregnancy and the first quarter of pregnancy was considered, the observed prevalence was similar. In our study, the two aforementioned intervals were also the ones with the highest prevalence of smoking cessation: 17% and 11%, respectively.

Although not significant in the crude analysis, in this study's adjusted analysis maternal age had an inverse association with cessation. Colman and Joyce^4^ also observed a higher prevalence of cessation among younger women in a study conducted in the United States (1993-1999). In a 1982 study carried out in Pelotas, Rio Grande do Sul, younger women were found to quit smoking in greater proportion. This tendency was not observed in 1993, when smoking cessation became more common in both age extremes^5^.

Socioeconomic levels have been shown to be associated with several risk factors and different morbidities. This association also occurs for smoking during pregnancy, with a higher prevalence of cessation among pregnant women with better socioeconomic levels. Thus, as observed in this study, the majority of the researches demonstrate that women with higher income are more likely to stop smoking during pregnancy^5^ ^,^ ^6^ ^,^ ^9^. Therefore, the health professionals’ focus should be on women at the lowest socioeconomic level.

In addition to the association with family income, the studied population had a high prevalence rate among parturients with at least nine years of schooling (PR = 2.8; 95%CI 1.27-6.29), which may indicate that this is an important feature in determining cessation during pregnancy. Besides higher socioeconomic levels, high maternal schooling may allow for greater access to information on the risks of smoking during pregnancy. The association with high schooling was also observed in three studies conducted in the United States, which gathered data from 1991^7^, 1993-1999^4^ and 2000-2005^9^.

The type of service in which the pregnant woman performed prenatal consultations (public or private) is not in itself a risk factor, but may be an indicator of exposure to other factors such as low prenatal care quality, lower income, and lower schooling. In the crude analysis, the prevalence of cessation among users of the private service was 46% higher than among users of the public service. The direction of this association was expected to remain equal in the adjusted analysis, but after adjustment it was inverted, with a 40% lower prevalence of cessation among women who used the private service. This finding is difficult to explain, and may be due to variables not evaluated here. It could be that women with lower income are now having consultations in the private sector, possibly due to the financial support of a relative, especially the partner. These pregnant women began to have access to this type of service due to the generation of thousands of jobs in the municipality of Rio Grande, as a result of new oil rigs being constructed. However, this hypothesis would need to be further investigated. This variable was not evaluated in other studies, but in the United States there was a higher prevalence of smoking during pregnancy among women assisted by Medicaid^20^ ^-^ ^22^ and greater cessation among those who were not assisted by this social program^9^.

Prenatal consultations increase the possibility of interventions by health professionals. In addition, a low number of consultations may indicate late initiation of prenatal care, unwanted pregnancy, and worse socioeconomic status. In this study, the prevalence of cessation was higher among women who performed at least six prenatal consultations. This is in agreement with other research findings, in which number of prenatal consultations^5^ or beginning prenatal care in the first quarter^6^ ^,^ ^9^ presented a directly proportional association with prevalence of cessation.

Here, women who did not smoke in the previous pregnancy had a prevalence of cessation almost three times higher. Among women who smoked in the previous pregnancy, only 12% stopped smoking in the current pregnancy. A study conducted in Norway (1999-2008) showed that 31% of women who smoked in the first pregnancy did not do so on the second^23^. Smoking in the previous pregnancy is indicative of longer smoking time and appears to be an important risk factor for carrying the habit into the subsequent pregnancy. In addition, the results presented here indicate that smoking time may be more important than the intensity of smoking (number of cigarettes smoked before pregnancy), since this variable lost statistical significance after adjustment.

When interpreting these data, one should keep in mind that it is comprised of information obtained from mothers’ reports, which may have led to an overestimation of smoking cessation prevalence. Kharrazi et al.^24^ showed that pregnant smokers tend to under-report smoking when answering “yes or no” questions, compared to multiple questions, which are able to better indicate changes in this behavior during pregnancy. In order to try to minimize the omission of smoking in this study, several questions were asked about the subject. The answers were expected to be coherent, allowing the investigation of smoking before and during pregnancy, as well as cessation attempts and withdrawal periods. Recent studies have shown that omission of smoking during pregnancy may be higher or lower depending on factors such as the cutoff used for the biochemical marker^25^, the type of questions^24^ and the moment of information collection^26^.

In any case, the prevalence of smoking cessation observed in this study was low. If it has indeed been overestimated, one has to conclude that the need for research on this subject is even greater. It is widely known that the gestational period is highly appropriate to encourage smoking cessation, since in this period women are more willing to change in this respect. Whenever the pregnant woman is in the health service, the health team, especially physicians and nurses, need to work more intensely and repeatedly with the issue of smoking cessation. In this sense, this study may offer an expressive contribution: it was able to identify the characteristics of women in the puerperal period who abandoned smoking and the moment when this behavior has a greater change of occurring. This makes it possible to direct the actions of health professionals, allowing their interventions to achieve greater impact.

Consideration should also be given to continuing actions for discouraging smoking after childbirth, in routine immunization visits, and for monitoring child development and growth through the fifth year of life. For many mothers, abstinence does not necessarily mean permanent cessation of smoking. To succeed, these interventions need to be repeated every time the mother is in the health services. Campaigns discouraging smoking could also be more intensively publicized by different types of media in order to reach these women at other times. Reducing the harmful impact of smoking on maternal and child health remains a major challenge to be overcome.
